# Thermoelectric properties of n-type Cu_4_Sn_7_S_16_-based compounds†

**DOI:** 10.1039/c9ra00077a

**Published:** 2019-03-08

**Authors:** Tingting Deng, Tian-Ran Wei, Qingfeng Song, Qing Xu, Dudi Ren, Pengfei Qiu, Xun Shi, Lidong Chen

**Affiliations:** State Key Laboratory of High Performance Ceramics and Superfine Microstructure, Shanghai Institute of Ceramics, Chinese Academy of Sciences Shanghai 200050 China wtr@mail.sic.ac.cn xshi@mail.sic.ac.cn; Center of Materials Science and Optoelectronics Engineering, University of Chinese Academy of Sciences Beijing 100049 China; School of Physical Science and Technology, ShanghaiTech University Shanghai 201210 China

## Abstract

Copper-based chalcogenides have ultralow thermal conductivity and ultrahigh thermoelectric performance, but most of them are p-type semiconductors. It is urgent to develop n-type counterparts for high efficiency thermoelectric modules based on these copper based-chalcogenides. Cu_4_Sn_7_S_16_ is an intrinsically n-type semiconductor with complex crystal structure and low thermal conductivity. However, its thermoelectric properties have not been well studied when compared to the well-known n-type CuFeS_2_. In this work, high-quality Cu_4_Sn_7_S_16_-based compounds are fabricated and their thermoelectric properties are systematically studied. Using Ag and Sb as dopants, the carrier concentration is tuned over a wide range. The electrical transport properties can be well described by the single parabolic band model with carrier acoustic phonons scattering. It is revealed that Cu_4_Sn_7_S_16_ exhibits a low effective mass and relatively high mobility. The thermal conductivity is lower than 0.8 W m^−1^ K^−1^ from 300 to 700 K and shows a weak dependence on temperature. A maximum *zT* of 0.27 is obtained in Cu_3.97_Ag_0.03_Sn_7_S_16_ at 700 K. Further enhancement of thermoelectric performance is possible when a more efficient n-type dopant is used.

## Abstract 2

Thermoelectric properties were optimized by Ag-doping in n-type Cu_4_Sn_7_S_16_-based compounds with complex structure, small effective mass and low thermal conductivity.

## Graphical abstract

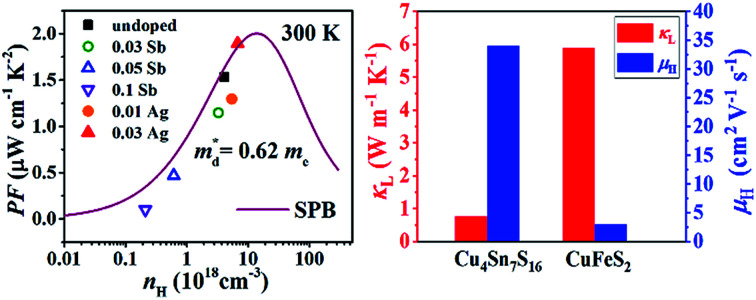

## Introduction

Thermoelectric (TE) materials are able to realize the direct conversion between heat and electricity. The conversion efficiency depends mainly on the dimensionless figure of merit *zT* = *S*^2^*σT*/*κ*, where *S* is the Seebeck coefficient, *σ* is the electrical conductivity, *T* is the absolute temperature and *κ* is the thermal conductivity.^1–4^ In recent years, Cu-based TE materials have emerged as promising thermoelectric materials not only for the large abundance and low-toxicity of the elements, but also for the diverse structure and lattice dynamics, interesting transport properties and high thermoelectric performance.^5–8^

It has been realized that most of the Cu-based TE materials are p-type semiconductors probably due to the intrinsic Cu vacancies that have a low formation energy,^9^ such as Cu_2_X (X = S, Se, Te),^10^ diamond-like compounds^11^ and their derivatives.^12^ However, a TE device is composed of both p-type and n-type legs. Also, the p- and n-type counterparts should match each other in electrical, thermal and mechanical properties and operation temperatures. Therefore, it is crucial to develop high-efficiency, compatible n-type Cu-based materials for application.

**Fig. 1:**
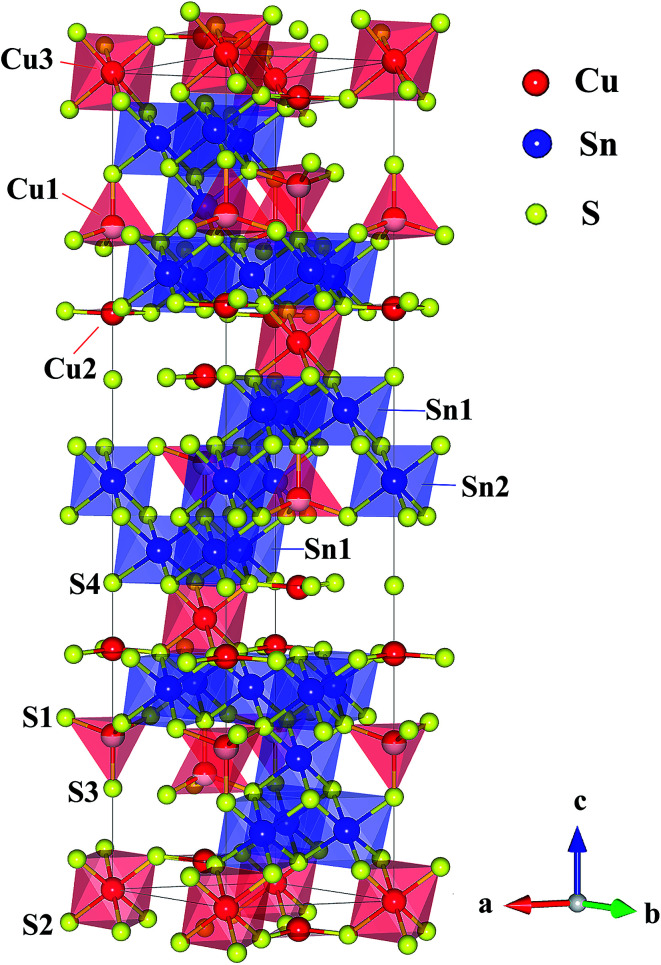
Crystal structure of Cu_4_Sn_7_S_16_. The crystallographic parameters are taken from ref. 19.

So far, there are only a few n-type Cu-based TE compounds reported probably due to the prevalent presence of intrinsic Cu vacancies. The current n-type copper chalcogenides are mainly ternary sulfides where S vacancies may compete with Cu vacancies, acting as donors. CuFeS_2_, *i.e.* the well-known chalcopyrite, exhibits a maximum *zT* of ∼0.2 with a high lattice thermal conductivity (∼5.9 W m^−1^ K^−1^) and a low carrier mobility (∼3.0 cm^2^ V^−1^ s^−1^).^13^ A lot of studies have been carried out on this compound, providing an insight into the crystal structure, phase variation, transport behaviour and underlying mechanisms as well as synthesis techniques.^13–16^ Another compound CuFe_2_S_3_ tends to decompose into CuFeS_2_ and Fe_7_S_8_ at room temperature.^17^ Besides, Cu_5_FeS_4_ exhibits an n–p transition around 450 K due to the intrinsic excitation.^18^ The compound Cu_4_Sn_7_S_16_, a largely distorted derivative of diamond-like materials, possesses a complex crystal structure (Fig. 1) with a very large cell volume 1694.8 Å^3^ and 81 atoms per unit cell,^19^ which is likely to result in a low lattice thermal conductivity favorable to thermoelectric performance.^20^

Previous studies^20,21^ have shown that Cu_4_Sn_7_S_16_ exhibits a pretty low thermal conductivity (∼1 W m^−1^ K^−1^ at 300 K) and high Seebeck coefficient varying from −200 to −750 μV K^−1^ depending on the synthesis processes, suggesting the potential of this compound as a promising n-type Cu-based TE material. In 2015, Bourgès *et al.* reported an optimized *zT* of 0.2 at 600 K.^20^ The main barrier limiting TE performance of Cu_4_Sn_7_S_16_ is its low electrical conductivity, about 10^−3^ to 10^3^ S m^−1^ at room temperature,^19–21^ which is far lower than conventional TE materials (10^4^ to 10^5^ S m^−1^).^22^ Therefore, feasible doping is needed to optimize the electrical properties, which has rarely been conducted. Recently, Cui *et al.* found that excess Sn and Se substitution for S can enhance *zT* up to 0.5 at a relatively high temperature of 873 K.^23,24^ Nonetheless, the carrier concentration is limited to the order of 10^16^ cm^−3^ in their work, and a deep insight into the electrical transports is needed based on the samples of a wide range of carrier density.

In this work, we synthesized high-purity Cu_4_Sn_7_S_16_-based compounds and studied their electrical and thermal transports as well as the doping effects. Using Sb and Ag as dopants, the carrier concentration was tuned from 2.1 × 10^17^ cm^−3^ to 6.7 × 10^18^ cm^−3^. The electrical transport properties can be reasonably described by the single parabolic band model. When compared with CuFeS_2_, the compound Cu_4_Sn_7_S_16_ was found to have a higher mobility due to the absence of d electrons from Fe and a lower thermal conductivity benefiting from the complex crystal structure. A maximum *zT* = 0.27 was achieved at 700 K in Ag-doped Cu_4_Sn_7_S_16_.

## Experimental

Pristine Cu_4_Sn_7_S_16_ and Sb-, Ag-doped samples were synthesized by the conventional melting-annealing method as described elsewhere.^20^ High-purity raw materials of Cu (shots, 99.999%, Alfa Aesar), Ag (shots, 99.999%, Alfa Aesar), Sn (shots, 99.999%, Alfa Aesar), Sb (shots, 99.999%, Alfa Aesar) and S (pieces, 99.999%, Alfa Aesar) were weighed out according to the stoichiometric ratio, and sealed in quartz tubes under vacuum in the glove box filled with argon. The tubes were put into the vertical furnace (NaberTherm). The furnace was heated to 723 K in 4.5 hours and held at this temperature for 2 hours, then heated to 1123 K in 4 hours and kept at this temperature for 2 hours. Then the temperature was decreased to 973 K in 1.5 hours and kept at this temperature for 2 days. The ingots were then fully ground in the agate mortar, pelletized, sealed into quartz tubes, and annealed at 973 K for 3 days. Finally, the products were ground into powders and sintered at 923 K under a uniaxial pressure of 65 MPa for 15 minutes by Spark Plasma Sintering (Sumitomo, SPS-2040). The sintering was carried out in argon atmosphere, and the pressure is about 0.07 MPa. The relative densities of all samples are around 99% measured by the Archimedes method.

The phase purity and crystal structure were detected by X-ray diffraction with Cu K_α_ sources (XRD, D/max-2550 V, Rigaku, Japan). Transmission electron microscopy (TEM, JEM-2100F, Japan) and selected area electron diffraction (SAED) were used to check the crystal structure. The distribution of chemical composition was characterized by scanning electron microscopy (SEM, ZEISS supra 55, Germany) and Energy Dispersive X-ray Spectroscopy (EDS, Oxford, UK) with accelerate voltage set as 20 kV. X-ray photoelectron spectroscopy (XPS, ESCALAB250, USA) was used to analyze the valence state of main elements in the samples, and the sample surface was sputtered by Ar^+^ beam before testing (2 kV, 20 s). The electrical conductivity (*σ*) and Seebeck coefficient (*S*) were measured from 300 K to 700 K by using ZEM-3 (Ulvac-Riko Japan). The Hall coefficient (*R*_H_) was measured at room temperature by Physical Property Measurement System (PPMS, Quantum Design, USA). The optical diffuse reflectance (*R*) was conducted on powder samples by using the UV-Vis spectrum (Shimadzu Spectroscope, UV-3101PC, Japan) at room temperature. The optical energy gap (*E*_g_) was obtained based on the Kubelka–Munk method by extrapolating (1 − *R*)^2^/2*R* to 0 as a function of *hυ* (where *hυ* is photon energy).^25^ The thermal conductivity was derived from the formula *κ* = *ρC*_p_*λ*, where *ρ* is the density, *C*_p_ is the isobaric heat capacity estimated according to the Dulong–Petit law, *λ* is thermal diffusivity measured by using the Laser flash method (LFA457, Netzsch, Germany). The velocity of sound (*v*) was measured by using the Advanced Ultrasonic measurement system (UMS, TECLAB, France).

## Results and discussion

Cu_4_Sn_7_S_16_ crystallizes into a rhombohedral structure with the space group of *R*3̄*m* (no. 166, *Z* = 3) as shown in Fig. 1, where sulfur atoms (S1, S2, S3 and S4) form the frameworks of tetrahedrons and octahedrons. Three kinds of octahedral sites are occupied by Sn1, Sn2 and Cu3, and two tetrahedral sites are totally and half occupied by Cu2 and Cu1, respectively. Also, it is suggested that Cu2 and Cu3 atoms exhibit a large displacement,^19,21^ possibly contributing to the low thermal conductivity.^20,26^

**Fig. 2:**
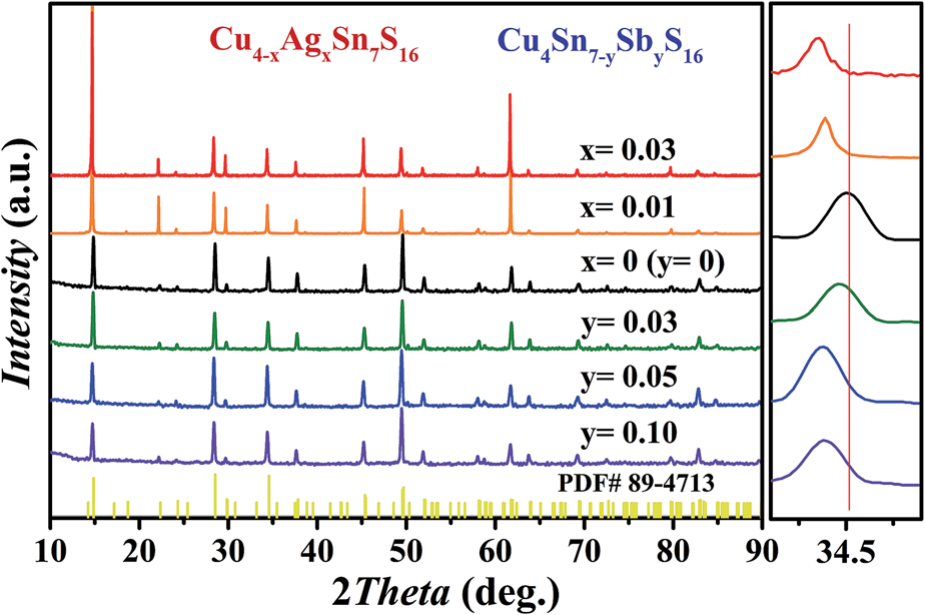
XRD patterns of Cu_4−*x*_Ag_*x*_Sn_7_S_16_ and Cu_4_Sn_7−*y*_Sb_*y*_S_16_ samples after SPS.

As shown in Fig. 2, all the samples were identified as Cu_4_Sn_7_S_16_ with the rhombohedral structure (*R*3̄*m*, PDF#89-4713) and no secondary phases were found when *x* ≤ 0.03 or *y* ≤ 0.10. Impurity phases emerge when the doping contents exceed these ranges (Fig. S1 and S2 in ESI†). The diffraction peaks shift slightly to the lower angles with increased Sb and Ag content. The Rietveld refinement results and the lattice constants (Fig. S3 and Table S1 in ESI†) confirm that the lattice volume expands upon Sb and Ag doping. These results indicate that the dopants have entered the lattice.

**Fig. 3:**
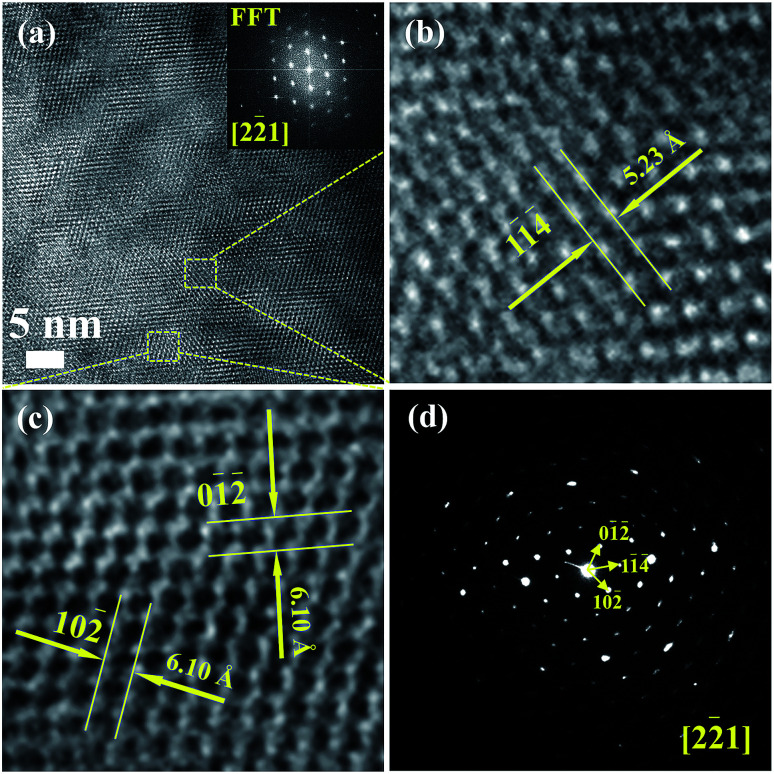
(a)–(c) HRTEM images for Cu_4_Sn_7_S_16_, and the inset picture of (a) is the fast Fourier transformation (FFT) image; (d) selected area electron diffraction pattern (SAED).

**Fig. 4:**
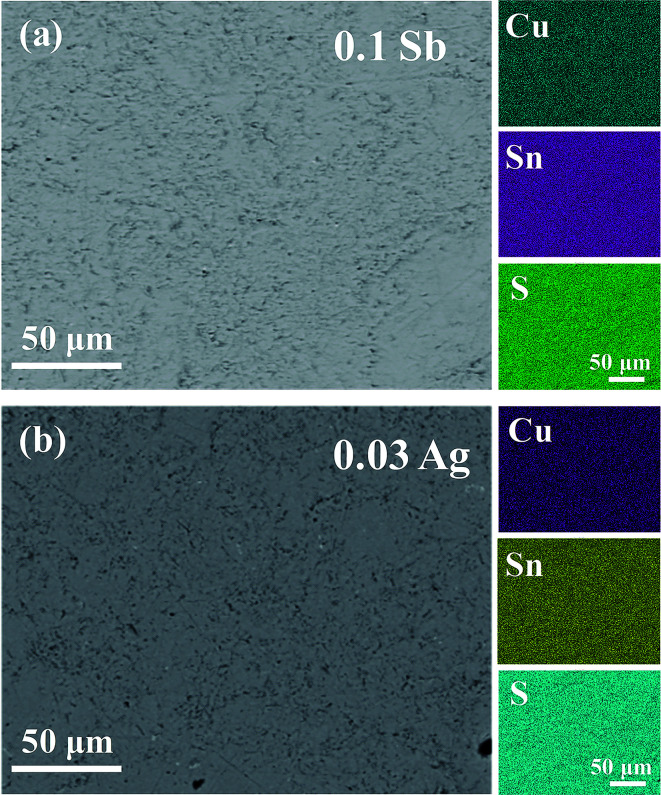
Backscattered electron (BSE) and EDS patterns of (a) Cu_4_Sn_6.9_Sb_0.1_S_16_ and (b) Cu_3.97_Ag_0.03_Sn_7_S_16_.

Fig. 3(a) shows the high-resolution TEM images of Cu_4_Sn_7_S_16_ and the inset is the fast Fourier transformation (FFT) for the entire area. The distances of crystal planes (01̄2̄), (11̄4̄) and (102̄) are 6.10 Å, 5.23 Å and 6.10 Å, respectively in Fig. 3(b) and (c), being well consistent with the standard diffraction data. The selected area electron diffraction pattern in Fig. 3(d) is clean and clear without any splitting, and agrees well with the FFT in Fig. 3(a), confirming the good crystallinity of the sample. It is worth mentioning that twinings were observed by Bourgès *et al.*^20^ Although we use the same sample fabrication process as Bourgès, the sintering temperature and holding time for final bulk samples are different. The difference in synthesis condition might lead to different microstructures and lattice defects. As shown in Fig. 4, the EDS images reveal that there are no visible impurities at micron scales, and the main elements exhibit a uniform distribution.

**Fig. 5:**
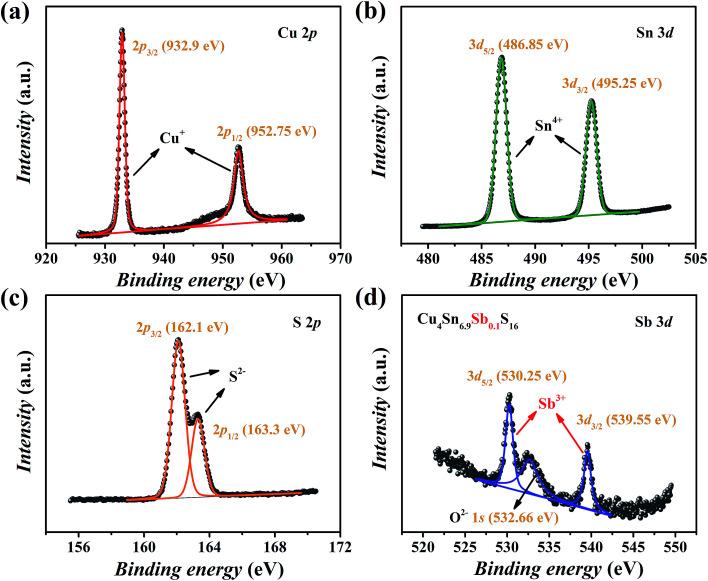
XPS spectra of Cu_4_Sn_6.9_Sb_0.1_S_16_: (a) Cu 2p, (b) Sn 3d, (c) S 2p and (d) Sb 3d.

The XPS spectra of Cu_4_Sn_6.9_Sb_0.1_S_16_ are shown in Fig. 5. The valence states of main elements Cu, Sn and S were identified as 1+, 4+ and 2−, respectively, which agrees with the available studies^19,23^ on Cu_4_Sn_7_S_16_. The binding energies of Sb were found to be 530.25 eV for 3d_5/2_ and 539.55 eV for 3d_3/2_ orbitals with a doublet separation (DS) of 9.3 eV (Fig. 5(d)), which is close to the data of Sb^3+^-containing materials such as Sb_2_S_3_ (BE: 529.6 eV for 3d_5/2_ and 539 eV for 3d_3/2_; DS: 9.4 eV),^27^ CuSbS_2_ (BE: 529.03 eV for 3d_5/2_ and 538.43 eV for 3d_3/2_; DS: 9.4 eV),^28^ and Cu_12_Sb_4_S_13_ (BE: 539.0 eV for 3d_3/2_).^29^ Therefore, the substitution of Sb^3+^ for Sn^4+^ is expected to generate extra holes.

**Fig. 6:**
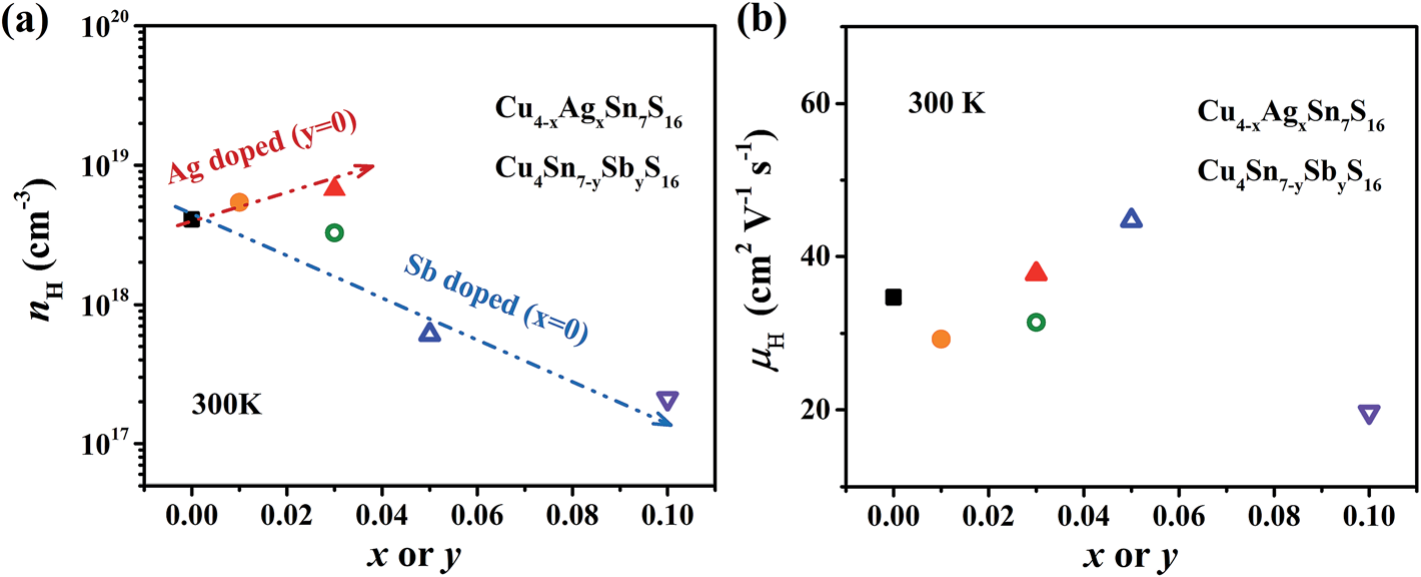
(a) Hall carrier concentration (*n*_H_) and (b) Hall carrier mobility (*μ*_H_) at room temperature against the contents of Ag and Sb. The arrows in (a) represent the trend of change.

The measured Hall carrier concentration *n*_H_ is shown in Fig. 6(a). *n*_H_ is 4.1 × 10^18^ cm^−3^ at 300 K for the undoped Cu_4_Sn_7_S_16_, being on the same order of magnitude of the reported data (9.2 × 10^18^ cm^−3^).^20^*n*_H_ decreases with increasing Sb content to 0.1, confirming that Sb is a p-type dopant as predicted by XPS. In contrast, Ag doping slightly enhances *n*_H_. This is in line with the common phenomena that Ag-based chalcogenides tend to show n-type conduction,^30–33^ which is probably related to the formation of more S vacancies or Ag interstitial atoms. The carrier mobility is 34 cm^2^ V^−1^ s^−1^ for undoped sample and does not change much when doped (Fig. 6(b)).

**Fig. 7:**
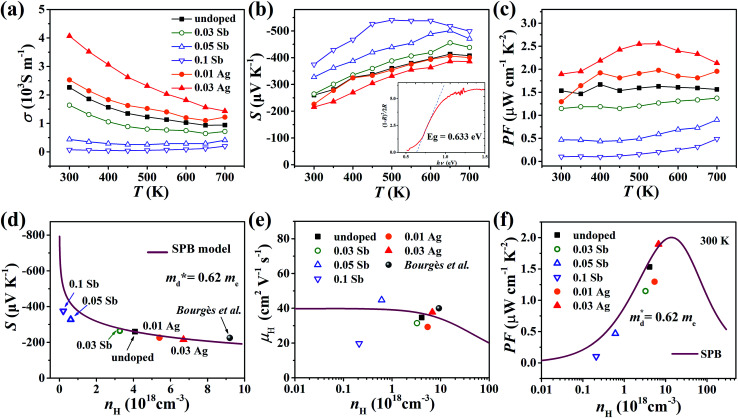
Temperature dependence of (a) electrical conductivity, (b) Seebeck coefficient and (c) power factor of all samples from 300 K to 700 K; the inset pattern in (b) represents the optical band gap of pristine Cu_4_Sn_7_S_16_. (d) Seebeck coefficient, (e) Hall mobility and (f) power factor varying with Hall carrier concentration at 300 K; the solid curves were calculated based on the single parabolic band (SPB) model with 
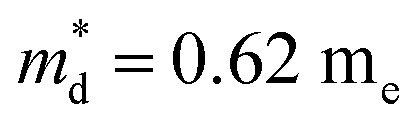
. The data of ref. 20 were also given for comparison.

The electrical conductivity of the pristine sample decreases from 2.3 × 10^3^ S m^−1^ at 300 K to 1 × 10^3^ S m^−1^ at 700 K as shown in Fig. 7(a). *σ* decreases when doped with Sb and increases with Ag, which agrees with the change of *n*_H_. All samples exhibit negative Seebeck coefficients in the entire measured temperature range, confirming the n-type conduction. The absolute values of *S* for all samples are relatively large, over 200 μV K^−1^, increasing with Sb content and decreasing with Ag content (Fig. 7(b)). According to the *E*_g_ = 2*eS*_max_*T*_*S*_max__ relation,^34^ the band gap was estimated to be 0.54 eV, which is reasonably consistent with the measured optical gap (0.63 eV, inset of Fig. 7(b)). The power factor (PF) changes in keeping with the carrier concentration (Fig. 7(c) and (f)).

In order to get an insight into the electrical transport properties of this compound, the single parabolic band (SPB) model was employed, and it was assumed that carriers are dominantly scattered by acoustic phonons. The transport parameters can be written as:^35^1
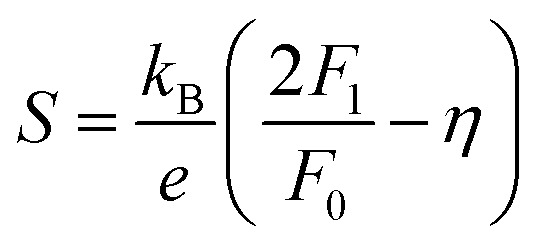
2
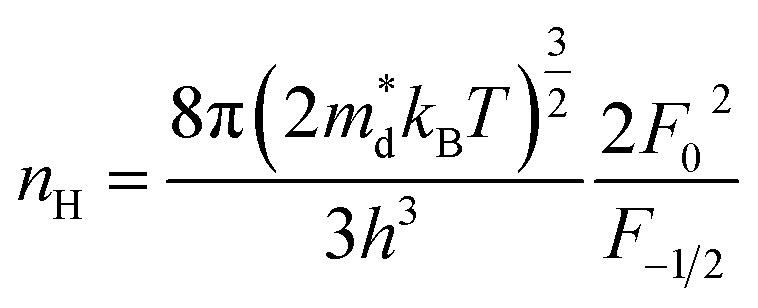
3
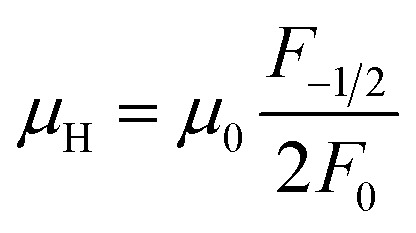

4 *PF* = *S*^2^*n*_H_*μ*_H_*e*

where *η* is the reduced Fermi level, *F*_i_(*η*) is the Fermi integrals expressed by 
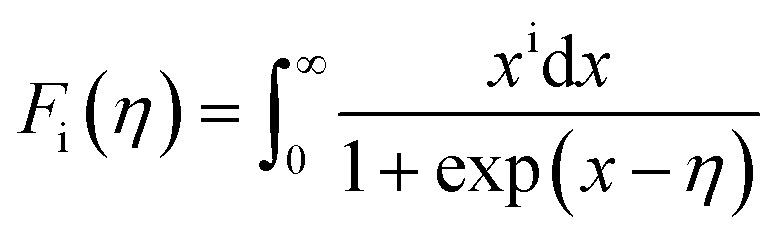
, 
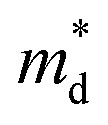
 is the density-of-state (DOS) effective mass, *k*_B_ is the Boltzmann constant, *h* is the Planck constant, *μ*_0_ is the mobility at the nondegenerate limit that was fitted to be 45 cm^2^ V^−1^ s^−1^. As shown in Fig. 7(d), the fitting line with 
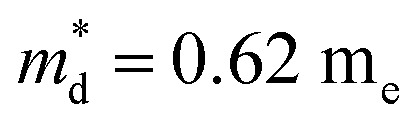
 was found adequate to describe the dependence of Seebeck coefficient on the carrier concentration. Such a small 
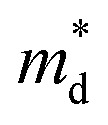
 is consistent with previous studies (∼0.73 m_e_),^20^ and may be related to the delocalized electrons from the sp orbitals of Sn. The small effective mass directly brings about a relatively high carrier mobility of 34 cm^2^ V^−1^ s^−1^ for undoped sample (Fig. 7(e)), being significantly larger than CuFeS_2_ (3.0–13.9 cm^2^ V^−1^ s^−1^).^13,14,16^ As shown in Fig. 7(f), the experimental data fall well on the theoretical line. The optimal carrier concentration is about 2 × 10^19^ cm^−3^ at room temperature, which is slightly higher than the one achieved in this work.

**Fig. 8:**
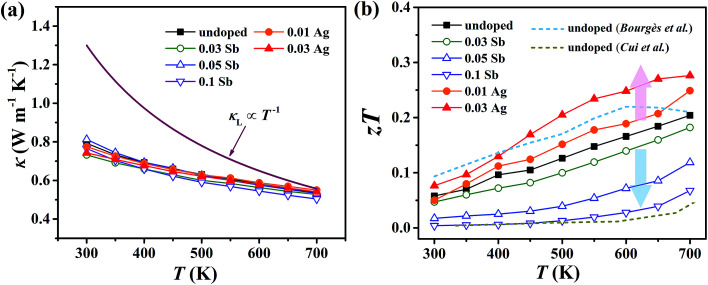
Temperature dependence of (a) total thermal conductivity (*κ*) and (b) figure of merit (*zT*). Blue and yellow dash lines represent the data taken from ref. 20 and 24, respectively.

For pristine Cu_4_Sn_7_S_16_, *κ* fluctuates from ∼0.8 W m^−1^ K^−1^ at room temperature (RT) to ∼0.55 W m^−1^ K^−1^ at 700 K (Fig. 8(a)). In contrast to the electrical properties, there is no obvious change in *κ* considering the small number of dopants. Due to the relatively low electrical conductivity, the lattice thermal conductivity *κ*_L_ accounts for >95% of the total *κ*. Such a low *κ*_L_ originates from the intrinsically complex crystal structure. Specifically, acoustic phonons are not only scattered by the Umklapp process (*κ*_L_ ∝ *T*^−1^),^36^ but also by the point defects (half occupation of Cu1 sites) and possible optical resonant modes arising from the large vibration of certain Cu atoms as widely found in Cu- and Ag-based chalcogenides.^33,37^ The relationship between the crystal structure, lattice dynamics, phonon dispersion and thermal transports will be comprehensively studied in the future. As shown in Fig. 8(b), *zT* is 0.18 at 700 K in undoped Cu_4_Sn_7_S_16_. When the content of Ag is 0.03 (Cu_3.97_Ag_0.03_Sn_7_S_16_), the optimal *zT* was enhanced to 0.27, which is 50% higher than the pristine one and also higher than the previous studies^20,23,24^ at the same temperature.

It is interesting to compare the two n-type Cu-based thermoelectric chalcogenides: Cu_4_Sn_7_S_16_ and CuFeS_2_. The elastic parameters of the two compounds were derived based on the measured sound velocity. The average sound velocity (*v*_s_) was calculated by^38,39^5
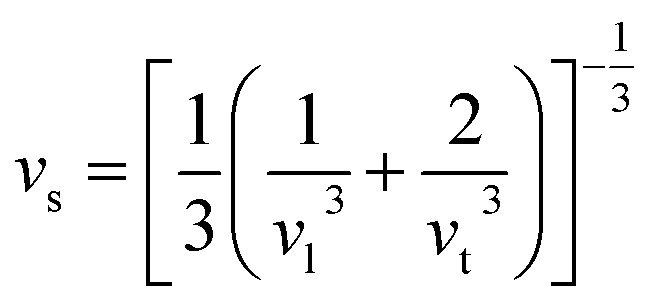
where *v*_l_ and *v*_t_ are the measured longitudinal and transverse velocity of sound (m s^−1^), respectively. The Debye temperature (*Θ*_D_) was calculated *via*^38,40^6
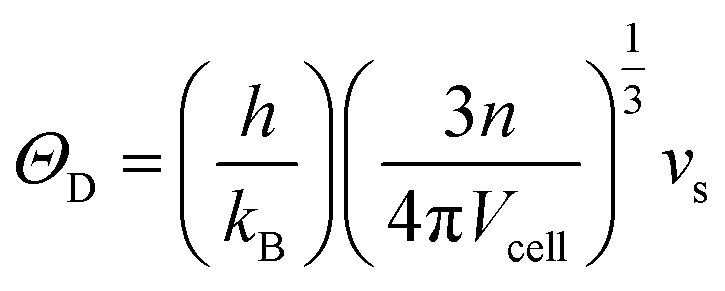
where *h* is the Planck constant, *k*_B_ is the Boltzmann constant, *n* is the number of atoms in the primitive unit cell, *V*_cell_ is the volume per unit cell. The Grüneisen parameter (*γ*) was estimated by^39,41,42^7
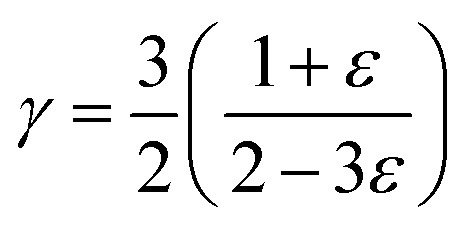
where *ε* is Poisson ratio calculated by the transverse and longitudinal velocity of sound (*v*_t_, *v*_l_)8
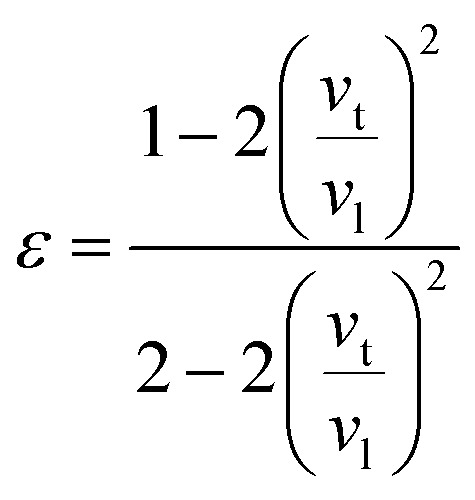


As shown in Table 1, the elastic parameters of the two compounds are comparable to each other, corresponding to the lowest lying acoustic phonon characters and intrinsic Umklapp process. Nonetheless, the *κ*_L_ of Cu_4_Sn_7_S_16_ is only 1/7 of that in CuFeS_2_, which originates from the complex crystal structure of the former. For electrical transports, the mobility of Cu_4_Sn_7_S_16_ is higher than CuFeS_2_ by an order of magnitude, which is probably related to the absence of valence electrons from the relatively localized d orbitals of Fe while the conduction band minimum (CBM) of CuFeS_2_ is mainly from the contribution of Fe 3d orbitals.^43^ The challenge for CuFeS_2_ lies in suppressing the *κ* while it is crucial to find more efficient n-type dopants for Cu_4_Sn_7_S_16_.

**Table 1:** Transport properties of n-type CuFeS_2_ and Cu_4_Sn_7_S_16_ materials at room temperature

	CuFeS_2_^a^	Cu_4_Sn_7_S_16_
Formula weight	183.53	1598.19
Space group	*I*4̄2*d*	*R*3̄*m*
*Z*	4	3
*V*_cell_ (Å^3^)	291.75	1697.9^c^
*d* (g cm^−3^)	4.19	4.70
*C*_p_ (J g^−1^ K^−1^)^b^	0.14	0.42
*v*_s_ (m s^−1^)	2293	2775
*v*_l_ (m s^−1^)	3764	4625
*v*_t_ (m s^−1^)	2056	2485
*Θ*_D_ (K)	259	300
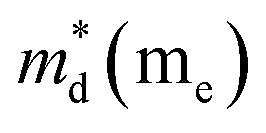	1.2	0.62
*γ*	1.7	1.8
*E*_g_ (eV)	0.34	0.63
*κ*_L_ (W m^−1^ K^−1^)	5.9	0.78
*σ* (S m^−1^)	2.3 × 10^3^	2.5 × 10^3^
*S* (μV K^−1^)	−370	−260
*n*_H_ (cm^−3^)	3.4 × 10^19^	4.0 × 10^18^
*μ*_H_ (cm^2^ V^−1^ s^−1^)	3.0	34

a Most of the parameters of CuFeS_2_ are taken from ref. 13.

b Estimated according to the Dulong–Petit law.

c Calculated by the Rietveld refinements.

## Conclusions

In this work, high-quality n-type Cu_4_Sn_7_S_16_-based compounds have been synthesized by the conventional melt-annealing method. The pristine material shows a large Seebeck coefficient and low electrical conductivity. The carrier concentration is tuned within a large range of 2.1 × 10^17^ to 6.7 × 10^18^ cm^−3^ by Ag- and Sb-doping. The electrical transports of Cu_4_Sn_7_S_16_ are well captured by the SPB model. Cu_4_Sn_7_S_16_ exhibits a low effective mass of 0.62 m_e_ and relatively high mobility of ∼34 cm^2^ V^−1^ s^−1^, which is ascribed to the delocalized character of sp orbitals and is in sharp contrast to 3d electrons of Fe in CuFeS_2_. The lattice thermal conductivity is lower than 0.8 W m^−1^ K^−1^ above room temperature, and shows a weak dependence on temperature. Maximum *zT* of 0.27 is obtained in Cu_3.97_Ag_0.03_Sb_7_S_16_ at 700 K. Considering the intrinsically low thermal conductivity and decent mobility, the performance of this compound can be further enhanced by more efficient doping. The findings and analyses in this work will promote the understanding on thermoelectric transports of Cu_4_Sn_7_S_16_-based compounds and the development of n-type Cu-based thermoelectric materials.

## Conflicts of interest

There are no conflicts to declare.

## Supplementary Material

RA-009-C9RA00077A-s001
