# Supplementary material for: Thermoelectric properties of n-type Cu4Sn7S16-based compounds
Source: RSC Adv. 2019 Mar 8;9(14):7826–32. doi: 10.1039/c9ra00077a (PMC9062621; doi:10.1039/c9ra00077a)
Supplement: RA-009-C9RA00077A-s001 [file RA-009-C9RA00077A-s001.pdf]

Electronic Supplementary Material (ESI) for RSC Advances.
This journal is © The Royal Society of Chemistry 2019

Supporting Information

Thermoelectric properties of *n*-type $Cu_4Sn_7S_{16}$-based compounds

Tingting Deng,[abc] Tian-Ran Wei,[*a] Qingfeng Song,[ab] Qing Xu,[abc] Dudi Ren,[a] Pengfei Qiu,[a] Xun Shi,[*a] Lidong Chen[a]

[a]State Key Laboratory of High Performance Ceramics and Superfine Microstructure, Shanghai Institute of Ceramics, Chinese Academy of Sciences, Shanghai 200050, China

[b]Center of Materials Science and Optoelectronics Engineering, University of Chinese Academy of Sciences, Beijing 100049, China

[c]School of Physical Science and Technology, ShanghaiTech University, Shanghai 201210, China

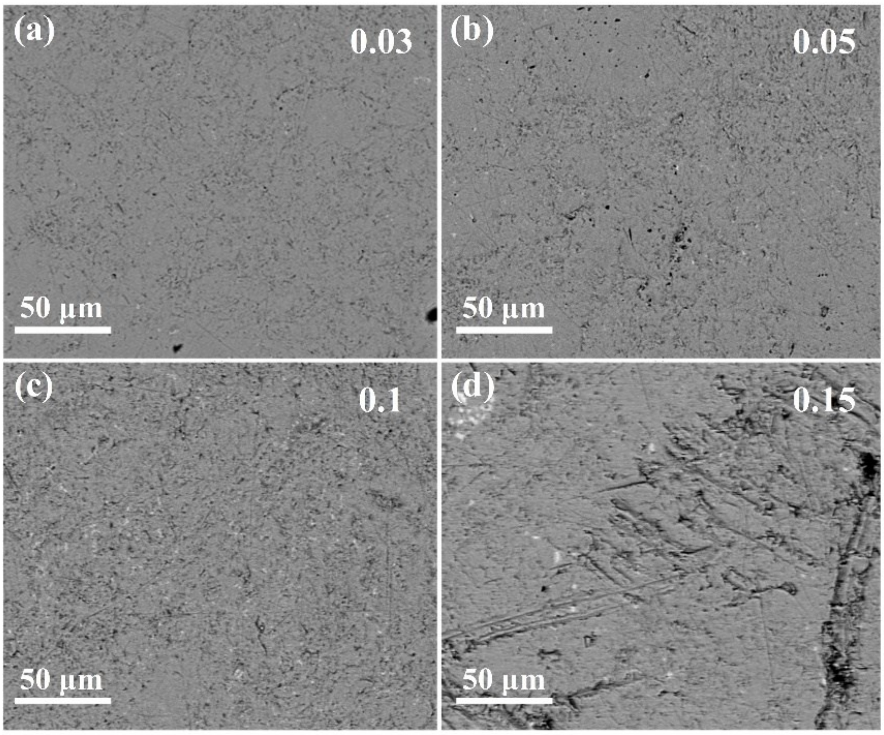

Fig. S1 Backscattered electron (BSE) patterns of $Cu_{4-x}Ag_xSn_7S_{16}$ (x= 0.03, 0.05, 0.1, 0.15). Impurity phases (white particles) emerge when *x* reaches 0.05.

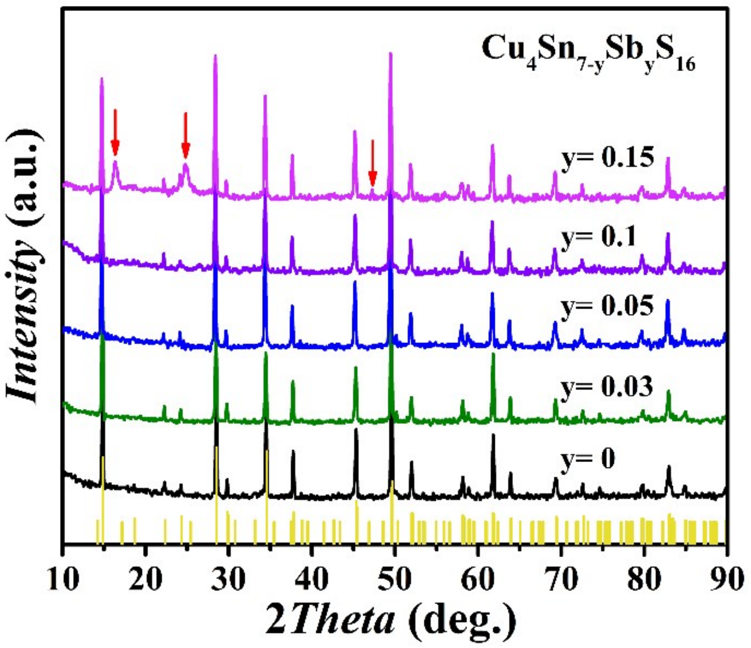

Fig. S2 XRD patterns of $Cu_4Sn_{7-y}Sb_yS_{16}$ (y= 0, 0.03, 0.05, 0.1, 0.15) bulks after SPS. Impurity phases emerge when *y* reaches 0.15.

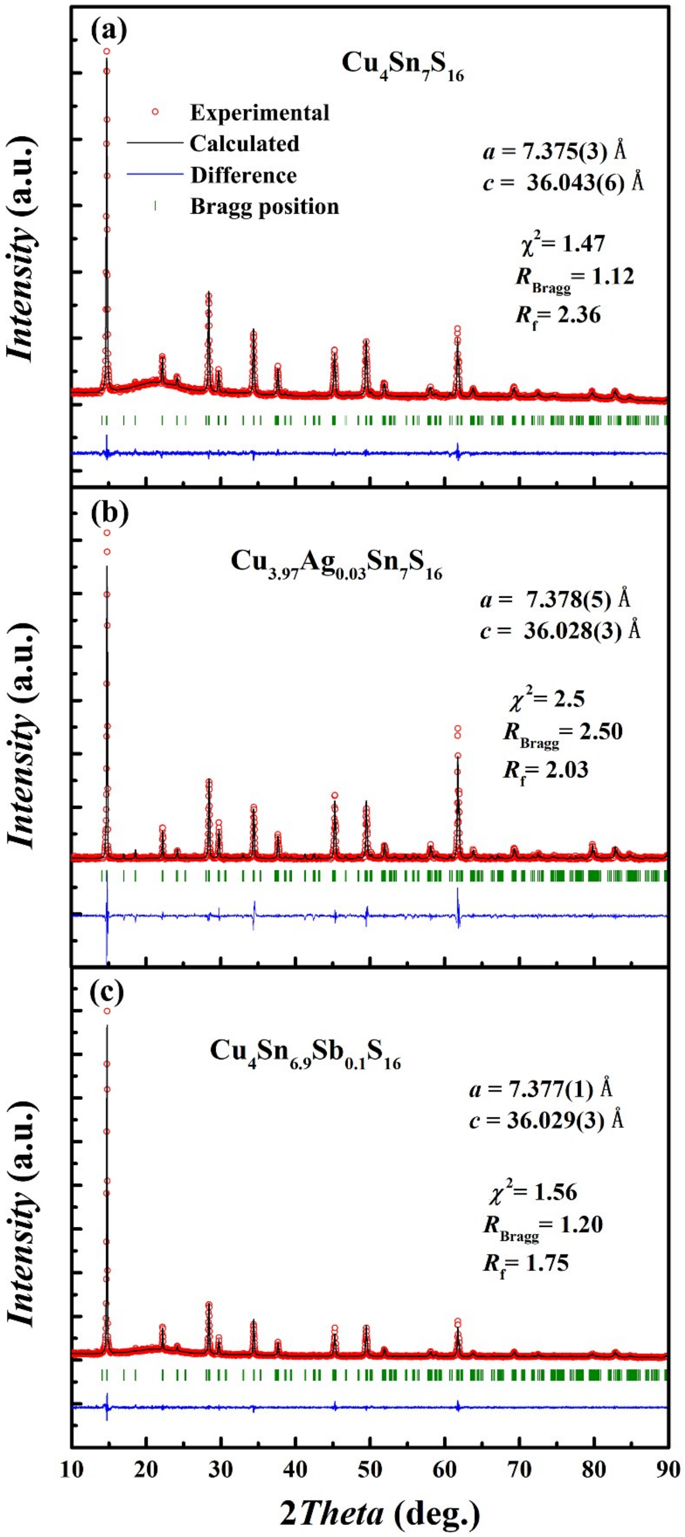

Fig. S3 Rietveld refinements for (a) $Cu_4Sn_7S_{16}$; (b) $Cu_{3.97}Ag_{0.03}Sn_7S_{16}$; (c) $Cu_4Sn_{6.9}Sb_{0.1}S_{16}$

Table S1 Refined lattice parameters for $Cu_4Sn_7S_{16}$, $Cu_{3.97}Ag_{0.03}Sn_7S_{16}$ and $Cu_4Sn_{6.9}Sb_{0.1}S_{16}$

	$Cu_4Sn_7S_{16}$	$Cu_{3.97}Ag_{0.03}Sn_7S_{16}$	$Cu_4Sn_{6.9}Sb_{0.1}S_{16}$
a (Å)	7.375	7.379	7.378
c (Å)	36.04	36.03	36.03
V_{cell} (Å^3)	1697.94	1698.69	1698.12
